# MetaSanity: an integrated microbial genome evaluation and annotation pipeline

**DOI:** 10.1093/bioinformatics/btaa512

**Published:** 2020-05-19

**Authors:** Christopher J Neely, Elaina D Graham, Benjamin J Tully

**Affiliations:** b1 Department of Biological Sciences, Los Angeles, CA 90089, USA; b2 Center for Dark Energy Biospheres Investigation, University of Southern California, Los Angeles, CA 90089, USA

## Abstract

**Summary:**

As the importance of microbiome research continues to become more prevalent and essential to understanding a wide variety of ecosystems (e.g. marine, built, host associated, etc.), there is a need for researchers to be able to perform highly reproducible and quality analysis of microbial genomes. MetaSanity incorporates analyses from 11 existing and widely used genome evaluation and annotation suites into a single, distributable workflow, thereby decreasing the workload of microbiologists by allowing for a flexible, expansive data analysis pipeline. MetaSanity has been designed to provide separate, reproducible workflows that (i) can determine the overall quality of a microbial genome, while providing a putative phylogenetic assignment, and (ii) can assign structural and functional gene annotations with varying degrees of specificity to suit the needs of the researcher. The software suite combines the results from several tools to provide broad insights into overall metabolic function. Importantly, this software provides built-in optimization for ‘big data’ analysis by storing all relevant outputs in an SQL database, allowing users to query all the results for the elements that will most impact their research.

**Availability and implementation:**

MetaSanity is provided under the GNU General Public License v.3.0 and is available for download at https://github.com/cjneely10/MetaSanity. This application is distributed as a Docker image. MetaSanity is implemented in Python3/Cython and C++. Instructions for its installation and use are available within the GitHub wiki page at https://github.com/cjneely10/MetaSanity/wiki, and additional instructions are available at https://cjneely10.github.io/year-archive/. MetaSanity is optimized for users with limited programing experience.

**Supplementary information:**

Supplementary data are available at *Bioinformatics* online.

## 1 Introduction

The analysis of microbial genomes has become an increasingly common task for many fields of biology and geochemistry. Researchers can routinely generate hundreds/thousands of environmentally derived microbial genomes using methodologies, such as metagenomics (Tully *et al.*, 2018), high-throughput culturing (Thrash *et al.*, 2015) and single-cell sorting (Stepanauskas *et al.*, 2017). However, analyzing the data can be problematic, as data analysis is computationally intensive and requires a knowledge of software that is constantly changing and may be difficult to install or execute. For the average researcher, the task of evaluating and annotating a set of microbial genomes may be time intensive and computationally rigorous. Here, we present MetaSanity, a comprehensive solution for generating evaluation and annotation pipelines for bacterial and archaeal isolate genomes, metagenome-assembled genomes (MAGs) and single-amplified genomes (SAGs). MetaSanity provides genome quality evaluation, phylogenetic assignment, as well as structural and functional annotation through a variety of integrated programs based on the procedure described in Tully (2019). By providing users with the ability to create annotation pipelines using multiple annotation suites, MetaSanity achieves a broad level of functional annotation that may be missed by implementing a single annotation program. MetaSanity provides a workflow that combines all outputs into a single queryable database that operates easily from the command line. Installation can be performed at the user level, limiting the need for intervention by system administrators, and, except for certain memory intensive programs, can be run locally on high-end personal computers.

## 2 Materials and methods

**Fig. 1.**
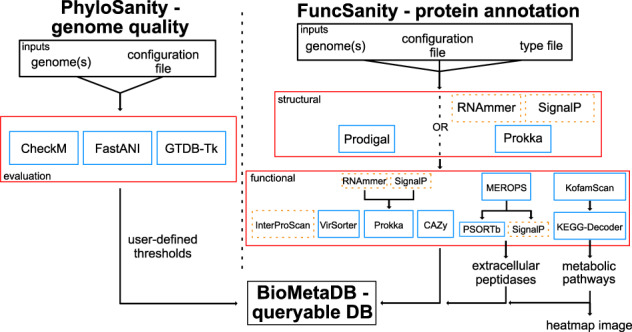
MetaSanity pipeline schema. Programs and databases that are part of the MetaSanity installation are in blue boxes. Programs in the dotted orange boxes must be installed separately by the user due to licensing agreements. DB, database. (Color version of this figure is available at *Bioinformatics* online.)

MetaSanity consists of two smaller workflows (Fig. 1): (i) PhyloSanity, to evaluate the completion, contamination, redundancy and phylogeny of each genome in a dataset, and (ii) FuncSanity, to provide structural and functional annotations of each genome. Each component consists of several optional applications that can be modified to specific research needs. While each component contained within the two pipelines runs independently and generates component-specific outputs, MetaSanity combines all outputs into a single queryable SQL database that allows fast and easy retrieval of data—in this case, gene annotations and other related genomic data. MetaSanity focuses on allowing users the ability to fine tune and adjust their data analysis pipelines with minimal effort and maximize computational and storage efficiency (Supplementary Table S1). MetaSanity is distributed as a Docker image (Merkel, 2014) and is implemented using a combination of Python3 (Python Software Foundation (2014)/Cython (Bradshaw *et al.*, 2011) and C++ (ISO/IEC 2014). MetaSanity is also available as a source code installation for systems that do not support Docker.

### 2.1 Phylosanity

PhyloSanity is designed to provide metrics of genome quality and to filter genomes for downstream analysis based on user-defined quality metrics. The workflow integrates CheckM v1.0.18 (Parks *et al.*, 2015), GTDB-Tk v.0.3.2 (Parks *et al.*, 2018) and FastANI (Jain *et al.*, 2018) as part of its evaluation pipeline. CheckM estimates the completion and contamination of each genome (Parks *et al.*, 2015). Next, FastANI compares each genome in a pairwise fashion against all other genomes to determine the average nucleotide identify (ANI) for each genome pair (Jain *et al.*, 2018). For any set of genomes that shares an ANI above a user-defined value, a non-redundant genome representative will be selected from the set that is the most complete and least contaminated. This allows users the option to exclude redundant genomes from further analysis. Differentiating genomes as non-redundant versus redundant can be useful for researchers working with MAGs or SAGs that are generated from replicate samples and may not have biological meaning when working with isolates or strain level differences. All genomes can undergo phylogenetic assignment based on relative evolutionary distance (Parks *et al.*, 2018) through GTDB-Tk (Pierre-Alain *et al.*, 2020), which will replace the CheckM-returned taxonomic assignment.

### 2.2 Funcsanity

FuncSanity provides structural and functional annotation of microbial genomes. The workflow incorporates annotation suites from eight existing and widely used programs. The use of multiple annotation programs has the advantage of capturing functional predictions that may not have been detected due to database or search limitations. Specialized annotation programs, such as VirSorter v1.0.5 (Roux *et al.*, 2015), use custom tools and/or databases to return relevant annotations that are not captured by other programs in MetaSanity. Open reading frames (ORFs) are predicted using Prodigal v2.6.3 (Hyatt *et al.*, 2010); however, users may opt to use the putative coding DNA sequences (CDS) generated by Prokka v1.13.3 (Seemann, 2014). From here, putative ORFs are processed by a set of annotation tools that can be selected by the user with user-defined filtering and cut-off values.

#### 2.2.1 Kyoto Encyclopedia of Genes and Genomes annotation

Putative ORFs can be searched against the KofamKOALA database using KofamScan v.1.1.0 (Aramaki *et al.*, 2019). Default parameters are used, and the ‘mapper’ tab-delimited output option is generated, linking ORF IDs to Kyoto Encyclopedia of Genes and Genomes (KEGG) Ontology (KO) IDs. Users can query any KO ID to generate specific functional search results in BioMetaDB.

#### 2.2.2 KEGG-Decoder

KEGG annotations can be used to estimate the completeness of various biogeochemically relevant metabolic pathways in a genome using KEGG-Decoder v.1.0.10 (Graham *et al.*, 2018; https://github.com/bjtully/BioData/tree/master/KEGGDecoder). Users can search genomes based on completeness of a pathway or function of interest. An additional heatmap summary visualization is generated.

#### 2.2.3 Virsorter

VirSorter v1.0.5 (Roux *et al.*, 2015) can be implemented to identify phage and prophage signatures in each genome using default parameters. Users can search for matches to each of the phage and prophage categories returned by VirSorter and generate lists of contigs and/or genomes with the assignments (Supplementary Table S1).

#### 2.2.4 Interproscan

InterProScan 5.36-75.0 (Jones *et al.*, 2014) is an optional installation and can be used for domain prediction on putative ORFs. Users have the option of downloading all of the InterProScan databases, including TIGRfam (Haft *et al.*, 2003), Pfam (Finn *et al.*, 2016), CDD (Marchler-Bauer *et al.*, 2010, Zhang *et al.*, 2017) and PANTHER (Mi *et al.*, 2019). Each InterProScan database result is indexed separately in BioMetaDB and can be used to return matching genomes using database specific IDs (e.g. PF01036 would return putative rhodopsin ORFs from a Pfam result).

#### 2.2.5 Prokka annotation

If not chosen as the option for structural annotation, genomes can be annotated using Prokka and its associated databases with the parameters –addgenes (adds the ‘gene’ feature to each CDS in the GenBank output format), –addmrna (adds the ‘mRNA’ feature to each CDS in the GenBank output format), –usegenus (use the genus-specific databases), –metagenome (improve gene predictions for fragmented genomes) and –rnammer [sets RNAmmer as the preferred rRNA prediction tool instead of Barrnap (http://www.vicbioinformatics.com/software.barrnap.shtml)]. rRNA identification with RNAmmer v.1.2 (Lagesen *et al.*, 2007) and signal peptide detection with SignalP v.4.1 (Nielsen, 2017) are optional installations. Users are given the option to use Prokka-derived putative CDS, which take into consideration the locations of RNA gene sequences (tRNA, rRNA, tmRNA and ncRNA) in their downstream analysis. These putative proteins are used in further analysis instead of the Prodigal-derived CDS, which are not aware of RNA boundaries.

#### 2.2.6 Carbohydrate-active enzyme annotation

Putative ORFs can be assigned a putative carbohydrate-active enzyme (CAZy) functionality (Cantarel *et al.*, 2009) based on the dbCANv2 database (Zhang *et al.*, 2018). ORFs are searched against dbCANv2 using HMMER v3.1b2 (Eddy, 2011) with the minimum score threshold set to 75 (-T parameter).

#### 2.2.7 Peptidase annotation

Putative ORFs can be assigned to a peptidase family using a set of HMMs that represent the MEROPS database (Rawlings *et al.*, 2014). PSORTb v.3.0 (Yu *et al.*, 2010) and SignalP can be optionally performed on MEROPS matches to determine if a putative enzyme is predicted to be extracellular. An extracellular assignment is made if PSORTb predicts ‘extracellular’ or ‘outer membrane’ localization or if PSORTb returns ‘unknown’ localization and SignalP predicts the presence of a signal peptide. Users can search for genes and genomes based on overall MEROPS annotations or by searching for specific designations (e.g. GT41 for glycosyl transferase family 41).

InterProScan, SignalP and RNAmmer are not automatically distributed with MetaSanity and require users to download their binaries separately and agree to their individual license requirements.

#### 2.2.8 Configuration adjustments

Each run of MetaSanity can be adjusted to the needs of the genome(s) being analyzed. One of the required parameters for MetaSanity if a configuration (config) file that sets which analyses will be performed on a genome or set of genomes, including optional settings (e.g. number of CPU threads, etc.). Config files allow users to determine specific pipelines of analyses, as needed, and provide a record which can be used for future genome sets for rapid re-deployment of analyses.

### 2.3 Biometadb

MetaSanity outputs consist of tab-delimited descriptions of genomic data, which can easily be analyzed by a variety of external tools. We additionally provide BioMetaDB, a specialized relational database management system tool that integrates modularized storage and retrieval of FASTA records with the metadata describing them. This application uses tab-delimited data files to generate table relation schemas via Python3. Based on SQLAlchemy v.1.3.7 (Bayer, 2012), BioMetaDB allows researchers to efficiently manage data from the command line by providing operations that include: (i) the ability to store information from any valid tab-delimited data file and to quickly retrieve FASTA records or annotations related to these datasets by using SQL-optimized command-line queries; and, (ii) the ability to run all CRUD operations (create, read, update, delete) from the command line and from python scripts. Output from both workflows is stored into a BioMetaDB project, providing users a simple interface to comprehensively examine their data (Supplementary Table S2). Users can query application results used across the entire genome set for specific information that is relevant to their research, allowing the potential to screen genomes based on returned taxonomy, quality, annotation, putative metabolic function or any combination thereof. More information on using BioMetaDB is available via the BioMetaDB GitHub (https://github.com/cjneely10/BioMetaDB).

## 3 Results

MetaSanity was tested on two separate systems—a personal computer with an Intel core i5-4570 CPU @ 3.20 GHz processor with 4 cores and 32 GB of RAM operating the Ubuntu 18.04.3 LTS Linux distribution, and an academic server with an Intel Xeon E7-4850 v2 @ 2.30 GHz processor with 96 cores and 1 TB of RAM operating the Ubuntu 18.04.3 LTS Linux distribution. Reduced options were calculated on the personal computer using all four available threads and preset parameter flags that skip memory-intensive processes. Complete options were calculated on the academic server using 10 threads and no parameters to reduce memory usage. Runtime results are available in Supplementary Table S3. The current architecture relies on sequential completion of time intensive processes, several of which are optional for users. Ongoing modifications that take advantage of parallelizing these processes should decrease the overall computation time.

## Supplementary Material

btaa512_Supplementary_Data
